# Supplementary material for: Malignant Brenner Tumor of the Ovary: A Systematic Review of the Literature
Source: Cancers (Basel). 2024 Mar 9;16(6):1106. doi: 10.3390/cancers16061106 (PMC10968811; doi:10.3390/cancers16061106)
Supplement: Supplementary file 1 [file cancers-16-01106-s001.zip › Supplementary Table S1.pdf]

Authors	Year	Age	Presentation	Side	Tumor size (cm)	Stage	CA-125 (U/ml)	Surgery	Adjuvant therapy	Recurrence	Interval to recurrence (mo)	Second line therapy	Follow-up (mo)	Outcome
Mackinlay	1956	64	Pain	Right	15	NM	NM	NM	NM	NM	NM	NM	6	DOD
Abel	1957	48	AUB, pan	Left	5	Ib	NM	HBSO	RT	No	NA	NA	2	ANED
Abel	1957	62	Vaginal bleeding, pain nausea, vomiting	Left	25	Ia	NM	HBSO	No	Yes	27	No	27	DOD
Reel	1958	84	Uterine bleeding, lower abd. enlargement, pressure symptoms	Left	18	NM	NM	LSO	NM	No	NA	NA	24	ANED
Marshall	1970	69	Asymptomatic	Left	10	NM	NM	HBSO	NM	NM	NM	NM	NM	NM
Miles and Norris	1972	*	**	NM	***	I	NM	HBSO	No	No	NA	NA	5	ANED
Miles and Norris	1972	*	**	NM	***	I	NM	SO	No	No	NA	NA	42	ANED
Miles and Norris	1972	*	**	NM	***	I	NM	HBSO	No	No	NA	NA	25	DOC
Miles and Norris	1972	*	**	NM	***	I	NM	SO	No	No	NA	NA	78	DOC
Miles and Norris	1972	*	**	NM	***	III	NM	BSO	No	Yes	NM	NM	3	DOD
Miles and Norris	1972	*	**	NM	***	I	NM	HBSO	No	Yes	NM	NM	13	DOD
Miles and Norris	1972	*	**	NM	***	I	NM	HBSO	RT	Yes	NM	NM	27	DOD
Toriumi and Ijima	1973	55	Uterine bleeding, abd. Distension	Left	20	NM	NM	LSO	NM	NM	NM	NM	NM	NM
Hull and Campbell	1973	52	Abd. enlargement, abd. discomfort, diarrhea	Left	15	NM	NM	HBSO	No	Yes	36	RT	47	DOD
Pratt-Thomas et al.	1976	59	Dyspnea, right pleural effusion	Right	14	NM	NM	BSO	RT	Yes	NM	NM	56	DOD
Beck et al.	1977	67	Acute urinary retention	Right	14	IV	NM	HBSO, excision of mesenteric nodules	No	No	NA	NA	10	DOD
Shafeek et al.	1978	23	Right hypochondrium swelling, diarrhea	Right	9	IIIC	NM	HBSO	No	No	NA	NA	1	DOD
Chiarelli et al.	1978	67	Abd. pain	Left	NM	NM	NM	HBSO	None (patient refused)	Yes	3	Inoperable tumor-biopsy	6	DOD
Hayden	1981	68	WT loss and increased abd. girth	Bilateral	6.5-6.5	IV	NM	HBSO, omental biopsy	NM	NM	NM	NM	NM	NM
Magrina et al.	1982	63	Vaginal bleeding	Right	10	Ic1	NM	HBSO	Alkeran + RT	No	NA	NA	56	ANED
Haid et al.	1983	78	Abd. pain	Bilateral	10 + 5	IIIc	NM	BSO, OM	Alkeran	Yes	9	R1: Doxorubicin, Cyclophosphamide R2: Hexamethylmelamine + Cyclophosphamide + Amethopterin + 5-FU	22	DOD
Chen	1984	73	Pelvic discomfort.	Bilateral	12 + 8	Ia	NM	HBSO	No	Yes	4	Vincristine, Actinomycin-D, Cytoxan	6	DOD
Roth and Czernobilsky	1985	42	Adnexal mass	Right	14	Ia1	NM	HBSO	NM	No	NA	NA	33	ANED
Roth and Czernobilsky	1985	74	Abd. mass	Left	13.5	Ia1	NM	HBSO	NM	No	NA	NA	7	ANED
Roth and Czernobilsky	1985	76	Vaginal bleeding, abd. mass	Right	17	Ic	NM	HBSO	NM	No	NA	NA	108	DOC
Roth and Czernobilsky	1985	65	Adnexal mass	Right	9	Ia2	NM	HBSO	NM	No	NA	NA	65	ANED
Seldenrijk et al.	1986	79	Vaginal bleeding, abd. mass, WT loss	Right	14	NM	NM	HBSO, OM	NM	No	NA	NA	7	ANED
Hayashi et al.	1987	67	Abd. pain, WT loss	Right	10	III	NM	HBSO, OM	NM	NM	NM	NM	NM	NM

Authors	Year	Age	Presentation	Side	Tumor size (cm)	Stage	CA-125 (U/ml)	Surgery	Adjuvant therapy	Recurrence	Interval to recurrence (mo)	Second line therapy	Follow-up (mo)	Outcome
Chen and Hoffman	1988	59	Abd. pain, tenderness	Left	6	IIIc	NM	BSO, OM	Alkeran	Yes	78	NM	78	AWD
Chen and Hoffman	1988	72	Vaginal bleeding	Left	15	IIIc	NM	HBSO, omental biopsy	Alkeran	Yes	69	NM	69	AWD
Chen and Hoffman	1988	69	Abd. pain	Bilateral	14 + 6	IIIc	NM	BSO	Cisplatin, adriamycin, cytoxan , hexamethyl melamine. 1x Alkeran x2*	Yes (2)	5 and 29	RT	30	DOD
Thirumavalavan et al.	1992	63	Abd. discomfort and swelling	Left	20	Ia	NM	HBSO	NM	NM	NM	NM	NM	NM
Joh et al.	1995	79	Abd. swelling, vaginal bleeding	Right	11	III	NM	HBSO	NM	Yes	NM	NM	3	DOD
Kataoka et al.	1995	67	Abd. pain, tenderness	Left	15	IIIc	NM	HBSO, OM	Cyclophosphamide, Mitomycin C 5-FU, x10 Cisplatin, Bleomycin x4	Yes	9	Cisplatin	55	DOD
Kataoka et al.	1995	51	Abd. tenderness	Right	13	Ia	120	RSO	Cisplatin, pepleomycin, and vinblastine	Yes	12 and 60	R1:intraperitoneal inoculation of Cisplatin Cisplatin analogue NK 121 x6 R2:Cisplatin + Etoposide x3	69	DOD
Ahr et al.	1997	77	WT loss and pelvic mass	Right	5	IV	Normal	BSO	None (patient poor status)	NM	NM	NM	3	AWD
Yamamoto et al.	1999	55	NM	Left	NM	Ia	265.3	HBSO	NM	Yes	42 and 50	OM, liver tumor, retroperitoneal LND bilateral ovarian vessel dissection. Cyclophosphamide + Doxorubicin + Cisplatin x6	NM	DOD
Yamamoto et al.	1999	70	Discomfort	Right	15	NM	NM	HBSO	Pepleomycin, Vincristin, Mitomycin C Cisplatin	No	NA	NA	20	ANED
Baizabal-Carvallo et al.	2010	56	Bifrontal headache, tinnitus, blurred vision and dizziness	Left	NM	IV	NM	No	None	NA	NA	NA	3	DOD
Dris et al.	2010	77	Abd. distension and pelvic pain	Left	16	Ic	294	HBSO, OM, appendectomy	NM	No	NA	NA	3	ANED
Roth et al.	2012	85	Abd. mass	Right	9	not staged	NM	RO	Platinum based	Yes	NM	NM	24	DOD
Gezginc¸, et al.	2012	55	Abd. distention	Left	5	IIIc	27	HBSO, OM, LND	TC x6	No	NA	NA	6	DOD
Gezginc¸, et al.	2012	55	Abd. pain	Right	8	Ic	Normal	HBSO, OM, LND	TC x8	No	NA	NA	84	ANED
Gezginc¸, et al.	2012	66	Bleeding	Left	6.5	IIIc	9.6	HBSO, OM, LND	TC x6	Yes	11	TC x6	68	ANED
Gezginc¸, et al.	2012	49	Abd. pain	Bilateral	4.5 + 3	Ib	Normal	HBSO, OM, LND	None	Yes	12	TC x6 + x7	52	ANED
Gezginc¸, et al.	2012	43	Abd. pain	Bilateral	8 + 2	Ia	12.5	HBSO, OM, LND	-	No	NA	NA	57	ANED
Gezginc¸, et al.	2012	65	Abd.l pain	Left	15	IIIc	208	HBSO, OM, LND	TC x6	Yes	36	TC x6	46	ANED
Gezginc¸, et al.	2012	79	Abd. distention	Left	7	IV	75	HBSO, OM, LND	TC x6	Yes	5	TC x6	5	ANED
Gezginc¸, et al.	2012	46	Pelvic mass	Right	16.5	Ic	25	HBSO, OM, LND	TC x6	Yes	34	TC x8 + x6	43	ANED
Gezginc¸, et al.	2012	46	Menstrual irregularity	Bilateral	15.5 + 5	Ib	Normal	HBSO, OM, LND	-	No	NA	NA	38	ANED
Gezginc¸, et al.	2012	46	Asymptomatic	Right	6.5	IV	67	HBSO, OM, LND	TC x6	Yes	21	TC x6	29	ANED

Authors	Year	Age	Presentation	Side	Tumor size (cm)	Stage	CA-125 (U/ml)	Surgery	Adjuvant therapy	Recurrence	Interval to recurrence (mo)	Second line therapy	Follow-up (mo)	Outcome
Gezginç et al.	2012	75	Abd. pain	Bilateral	12 + 10	IIIc	448	HBSO, OM, LND	TC x6	No	NA	NA	26	ANED
Gezginç et al.	2012	49	Pelvic mass	Bilateral	10 + 11	IIIc	135	HBSO, OM, LND	TC x6	No	NA	NA	25	ANED
Gezginç et al.	2012	50	Abd. pain	Bilateral	4 + 2.5	IIIc	44	HBSO, OM, LND	TC x6	Yes	18	TC x6	20	ANED
Verma et al.	2012	60	Abd. pain	Right	8	NM	4,073.3	HBSO, OM, LN biopsy	Cisplatin, Cyclophosphamide, Adriamycin x6	No	NA	NA	6	ANED
St Pierre-Robson et al.	2013	53	Abd. bloating	Left	7.5	Ia	NM	HBSO	NM	NM	NM	NM	22	ANED
St Pierre-Robson et al.	2013	57	Abd. fullness	Left	13	Ia	99	LSO, omental sampling	NM	NM	NM	NM	24	ANED
St Pierre-Robson et al.	2013	68	NM	Left	17	Ia	NM	HBSO,OM	NM	NM	NM	NM	NM	NM
Han et al.	2014	37	Abd. pain, vaginal bleeding	Right	8	Ia	35.5	HRSO, OM, appendectomy, PPLND	None	No	NA	NA	26	lost
Han et al.	2014	42	Vaginal bleeding	Left	3	Ia	21.1	HBSO, OM, appendectomy, PPLND	None	No	NA	NA	155	lost
Han et al.	2014	59	Abd. pain	Right	2.5	IV	10.7	HBSO, OM, appendectomy, PPLND	neoadj. Cisplatin, Taxol x5, TC x6	Yes	9	Hycamtine-Cisplatin x6	173	ANED
Han et al.	2014	52	Abd. pain	Right	10.5	IIIc	8.3	HBSO, OM, appendectomy, PPLND	TC x9; Hycamtine-Carboplatin x1; Camptobell, Carboplatin x2; Gemcitabine-TC x6	Yes	18	NM	77	DOD
Han et al.	2014	61	Abd. pain	Right	13.5	Ic	23.4	HBSO, OM, appendectomy, PPLND	TC x6	No	NA	NA	21	DOC
Han et al.	2014	43	Abd. pain	Bilateral	7.5 + 6.5	IIc	724	HBSO, OM, appendectomy, PPLND	TC x6; Camptobell x6; Gemcitabine x3; Taxol x6; Beva x10; oral Etoposide; RT; Adriamycin x3; Etoposide; Beva x6	Yes	9	NA	101	DOD
Han et al.	2014	59	Abd. pain, mass	Right	25	Ia	13	HBSO, OM, appendectomy, PPLND	None	No	NA	NA	102	ANED
Han et al.	2014	68	Abd. pain, mass	Right	12.5	Ia	38.5	LSO	TC x6	No	NA	NA	8	lost
Han et al.	2014	48	Asymptomatic	Right	5.5	IIIc	4	HBSO, OM, appendectomy, PPLND	TC x6	Yes	13	TC x6; docetaxel-cisplatin x1	32	AWD
Han et al.	2014	61	Mass	Left	12	Ia	10.7	HBSO, OM, appendectomy, PPLND	None	No	NA	NA	16	ANED
Di Donato et al.	2016	46	AUB, abdominal pain	Right	9	IIIc	77.8	HBSO, OM, appendectomy, PPLND	TC	No	NA	NA	29	ANED
Yue et al.	2016	51	Abd. pain	Right	25	Ic	53.78	HBSO, OM, appendicectomy	Taxol + Cisplatin	No	NA	NA	38	DOD
Yue et al.	2016	56	Abd. distension	Right	NM	IIIc	143	HBSO, OM, appendicectomy	Taxol + Cisplatin	Yes	13	Ifosfamide + Nedaplatin ×8	10	DOD
Yue et al.	2016	43	Pelvic mass	Right	19	IV	45.69	RO	Cisplatin + Epirubicin	Yes	5	Taxol + Cisplatin ×1	34	DOD
Yue et al.	2016	55	Abd. pain	Right	NM	Ic	222.4	HBSO, OM	Oxaliplatin, Taxol ×7; Gemcitabine, Nedaplatin, ×4; TC ×3;	Yes	22	Docetaxel + Lobaplatin ×4	61	ANED

Authors	Year	Age	Presentation	Side	Tumor size (cm)	Stage	CA-125 (U/ml)	Surgery	Adjuvant therapy	Recurrence	Interval to recurrence (mo)	Second line therapy	Follow-up (mo)	Outcome
									Pemetrexed, Cisplatin ×1					
Yue et al.	2016	44	Abd. distension	Left	15	Ia	16.07	HBSO, OM, appendicectomy	Taxol + Cisplatin × 3	No	NA	NA	5	DOD
Yue et al.	2016	32	Hematuresis	Left	NM	IV	NA	HBSO, OM, bladder-involved focus resection	Cytoxan, Vincristine, Fluorouracil, BCNU ×1	NA	NA	NA	14	ANED
Yue et al.	2016	46	Abd. distension	Right	11	IIIc	356.07	HBSO, OM	Taxol, DDP ×2	Yes	4	DDP, Cytoxan, Fluorouracil ×3	46	ANED
Yue et al.	2016	48	Abd. distension, vaginal bleeding	Left	24	Ic	NA	HBSO, OM	Ifosfamide, Fluorouracil ×2	No	NA	NA	93	ANED
Yue et al.	2016	47	Pelvic mass	Bilateral	6 + 6	Ib	NA	HBSO, OM	CTX, VCR, 5-FU ×2	No	NA	NA	94	DOC
Yue et al.	2016	76	Vaginal bleeding, pelvic mass	Left	15	Ic	NA	HBSO, OM	None	No	NA	NA	94	DOC
Turgay et al.	2017	49	Abd. pain, mass	Bilateral	14	IIIc	51	HBSO, OM, PPLND, splenectomy, appendectomy	TC x6	No	NA	NA	24	ANED
Turgay et al.	2017	62	Abd. pain, mass	Right	18	IIIc	24	HBSO, OM, PPLND, appendectomy	TC x6	No	NA	NA	18	ANED
Toboni et al.	2017	54	Gastrointestinal complaints, abd. pain	NM	NM	NM	675	HBSO, OM	Platinum, Taxane x6	Yes (4)	48	R1: Surgery, taxane, platinum x6; R2: NA; R3: NA; R4: carboplatin	NA	AWD
Lang et al.	2017	77	Pelvic mass	Right	>10	IIc	14	RSO, PPLND	TC x6	Yes	12	TC x2; Carboplatin, Paclitaxel, Beva x10; Beva x8; Surgery; RT	24	ANED
King et al.	2018	58	vaginal bleeding, abd. fullness increasing urinary pressure and frequency	Right	25	NM	19.8	HBSO, OM, LND	None	No	NA	NA	2	ANED
King et al.	2018	79	Pelvic mass, abd. distension, pelvic discomfort, WT loss		25	NM	563.5	BSO	None	No	NA	NA	24	ANED
Agius et al.	2018	70	Abd. mass, WT loss, and constipation.	Left	18	IVb	11.13	HBSO, OM	Taxol, Cisplatin	No	NA	NA	NM	ANED
Zhang et al.	2019	77	AUB	NM	****	IIb	43	HBSO, OM LND	TC x5	Yes	116	No	117	DOD
Zhang et al.	2019	58	Pelvic pressure	NM	****	Ia	12.6	HBSO, OM, LND	TC x6	No	NA	NA	42	ANED
Zhang et al.	2019	60	Abd. pain	NM		IIb	91.7	BSO, OM, LND	TC x6	Yes (2)	12	R1 Doxorubicin, Carboplatin x3 + RT; R2 Tamoxifen, Gemcitabine x4, Eribulin x5	12	AWD
Zhang et al.	2019	67	Abd. pain	NM		IIa	25.4	BSO, OM, LND	TC x6	No	NA	-	5	ANED
Zhang et al.	2019	39	Abd. pain	NM		IVb	494.8	HBSO, OM	Carboplatin-Docetaxel x6	Yes (3)	17	R1: surgery + RT; R2: unknown; R3 Cyberknife	45	DOD
Zhang et al.	2019	70	Asymptomatic	NM		Ic1	10.5	HBSO, OM	TC x6	No	NA	NA	37	ANED
Zhang et al.	2019	69	AUB, abd. pain	NM		Ib	264	HBSO, OM, LND	TC x6	No	NA	NA	78	ANED
Zhang et al.	2019	82	Abd. pain	NM		IIIb	NA	HBSO, OM	neoadj. TC x6	Yes	NM	NM	28	AWD (lost)
Zhang et al.	2019	58	Pelvic pressure	NM		Ia	9.1	HBSO	None	No	NA	NA	126	ANED

Authors	Year	Age	Presentation	Side	Tumor size (cm)	Stage	CA-125 (U/ml)	Surgery	Adjuvant therapy	Recurrence	Interval to recurrence (mo)	Second line therapy	Follow-up (mo)	Outcome
Zhang et al.	2019	49	Abd. pain	NM		Ia	10.8	HBSO, OM, LND	NM	NM	NM	NM	NM	lost
Toshniwal et al.	2020	65	postmenopausal bleeding, abd. fullness	Right	14.1		227	HBSO,	TC	No	NA	NA	NM	NM
Singh et al.	2020	62	Abd. pain, vomiting and constipation	Right	10.2	IIIc	184.2	HBSO, right hemicolectomy	None	No	NA	NA	-	DICU
Bouhani et al.	2020	73	Abd. distension	Left	15	IIc	294		TC x6	Yes	9	Symptomatic treatment	14	AWD
Bouhani et al.	2020	46	Abd. pain	Left	9	IIIc	490		TC x4, Endoxan-Carboplatin x2	Yes (2)	11 and 31	R1 Gemzar-Adriamycin x6; R2 Paclitaxel x6	39	AWD
Bouhani et al.	2020	60	Pelvic mass	Right	8	IIIc	273.4		TC x6	Yes (2)	11 and 18	R1 TC x4, Surgery; R2 symptomatic treatment	64	AWD
Bouhani et al.	2020	58	Pelvic pain	Left	18	Ic	NA		TC x6	Yes	59	TC x1	59	lost
Wang et al.	2020	71	Vaginal bleeding, abd. pain	Right	20	Ia	NM	HBSO, peritoneal, omental biopsies	None	No	NA	NA	18	ANED
Mc Ginn et al.	2021	22	Asymptomatic	Left	11	Ia	Normal	LSO	None	Yes	50	NM	NM	NM
Mc Ginn et al.	2021	60	Asymptomatic	Left	4.5	Ia	NM	BSO	chemotherapy	No	NA	NA	14	ANED
Yüksel et al.	2022	75	Adnexal mass	Bilateral	3.6	IIIc	20	NM	TC x6	No	NA	NA	47	ANED
Yüksel et al.	2022	57	Adnexal mass	Left	5.5	IIa	NA	NM	TC x7 +RT	No	NA	NA	12	NM
Yüksel et al.	2022	48	Adnexal mass	Left	20	Ia	9.6	NM	None	No	NA	NA	96	ANED
Yüksel et al.	2022	37	Adnexal mass	Right	30	Ic1	12	NM	TC x6	No	NA	NA	115	ANED
Yüksel et al.	2022	49	Adnexal mass	Right	NA	IIIc	NA	NM	TC x6	Yes	86	Surgery; TC x6	96	ANED
Yüksel et al.	2022	75	Adnexal mass	Right	NA	IIIc	95	NM	TC x6	No	NA	NA	12	DOD
Yüksel et al.	2022	36	Adnexal mass	Right	18	Ic3	209	NM	TC x6	No	NA	NA	125	ANED
Yüksel et al.	2022	55	Adnexal mass, abd. pain	Bilateral	15	IIIc	64	NM	TC x6	Yes	13	Bleomycin, Etoposid, Cisplatin; RT	53	DOD
Zou et al.	2022	50	Abd. distension, pain	Bilateral	15.2-6.2	IIIc	256.3	HBSO, OM, LND	TC +RT	No	NA	NA	12	ANED
Kurniadi et al.	2023	39	Abd. mass, WT loss	Right	25	IIIa	NA	HBSO, OM, LND	TC x3	No	NA	NA	3	ANED

Table S1: Clinicopathologic and treatment features of the cases of malignant Brenner tumor.

Abbreviations: Abd.: Abdominal; AUB: Abnormal uterine bleeding; Beva: Bevacizumab; CHT: chemotherapy; HBSO: hysterectomy and bilateral salpingo-oophorectomy; OM: omentectomy; LND: lymph node dissection; NA: not applicable; NM: not mentioned; PPLND: pelvic and paraortic lymph node dissection; RO: Right oophorectomy; TC: Taxol-Carboplatin; WT: weight; *:38 to 87 (median 68); **: Unilateral adnexal mass: 5 patients; abdominal pain: 6 patients; abdominal distention: 5 patients; vaginal bleeding: 3 patients; nausea or vomiting: 4 patients; asymptomatic: 1 patient. ***: 11 to 22 cm (median 14.8cm). ****:6.5 to 25 cm in largest dimension, with a mean of 13.9 cm (stdev±6.5 cm).
