# Supplementary material for: Malignant Brenner Tumor of the Ovary: A Systematic Review of the Literature
Source: Cancers (Basel). 2024 Mar 9;16(6):1106. doi: 10.3390/cancers16061106 (PMC10968811; doi:10.3390/cancers16061106)
Supplement: Supplementary file 1 [file cancers-16-01106-s001.zip › Supplementary Table S2.pdf]

Supplement

Symptom	N	%
**	7	6.19
AUB	1	0.88
AUB, abd. pain	2	1.77
AUB, lower abd. enlargement, pressure symptoms	1	0.88
AUB, pain	1	0.88
Abd. bloating	1	0.88
Abd. discomfort and swelling	1	0.88
Abd. distension	4	3.54
Abd. distension and pelvic pain	1	0.88
Abd. distension, pain	1	0.88
Abd. distension, vaginal bleeding	1	0.88
Abd. distention	2	1.77
Abd. enlargement, abd. discomfort, diarrhea	1	0.88
Abd. fullness	1	0.88
Abd. mass	2	1.77
Abd. mass, WT loss	1	0.88
Abd. mass, WT loss, and constipation.	1	0.88
Abd. pain	22	19.47
Abd. pain, WT loss	1	0.88
Abd. pain, mass	4	3.54
Abd. pain, tenderness	2	1.77
Abd. pain, vaginal bleeding	1	0.88
Abd. pain, vomiting and constipation, anorexia, WT loss	1	0.88
Abd. swelling, vaginal bleeding	1	0.88
Abd. tenderness	1	0.88
Acute urinary retention	1	0.88
Adnexal mass	9	7.96
Adnexal mass, abd. pain	1	0.88
Asymptomatic	6	5.31
Bifrontal headache, tinnitus, blurred vision and dizziness	1	0.88
Bleeding	1	0.88
Discomfort	1	0.88
Dyspnea, right pleural effusion	1	0.88
Gastrointestinal complaints, abd. pain	1	0.88
Hematuresis	1	0.88
Increased abd. girth, WT loss	1	0.88
Mass	1	0.88
Menstrual irregularity	1	0.88
Pain	1	0.88
Pelvic discomfort.	1	0.88
Pelvic mass	6	5.31
Pelvic mass, WT loss	1	0.88
Pelvic mass, abd. distension, pelvic discomfort, WT loss	1	0.88
Pelvic pain	1	0.88
Pelvic pressure	2	1.77
Right hypochondrium swelling, diarrhea	1	0.88
Uterine bleeding, abd. Distension	1	0.88
Vaginal bleeding	3	2.65
Vaginal bleeding, abd. mass	1	0.88
Vaginal bleeding, abd. mass, WT loss	1	0.88
Vaginal bleeding, abd. pain	1	0.88
Vaginal bleeding, pain, nausea, vomiting	1	0.88
Vaginal bleeding, pelvic mass	1	0.88
Postmenopausal bleeding, abd. fullness	1	0.88
Vaginal bleeding, abd. fullness, increasing urinary pressure, and frequency	1	0.88

Table S2: Symptoms at presentation

Abbreviations: Abd: abdominal; AUB: abnormal uterine bleeding; WT: weight.
