# Supplementary material for: Malignant Brenner Tumor of the Ovary: A Systematic Review of the Literature
Source: Cancers (Basel). 2024 Mar 9;16(6):1106. doi: 10.3390/cancers16061106 (PMC10968811; doi:10.3390/cancers16061106)
Supplement: Supplementary file 1 [file cancers-16-01106-s001.zip › Supplementary Table S3.pdf]

Stage	N	Subtotal	%
**I**	6	50	50%
**Ia**	22		
**Ia1**	2		
**Ia2**	1		
**Ib**	5		
**Ic**	10		
**Ic1**	3		
**Ic3**	1		
**IIa**	2	7	7%
**IIb**	2		
**IIc**	3		
**III**	3	32	32%
**IIIa**	1		
**IIIb**	1		
**IIIc**	27		
**IV**	9	11	11%
**IVb**	2		

Table S3: Details from tumor staging
