# Supplementary material for: Malignant Brenner Tumor of the Ovary: A Systematic Review of the Literature
Source: Cancers (Basel). 2024 Mar 9;16(6):1106. doi: 10.3390/cancers16061106 (PMC10968811; doi:10.3390/cancers16061106)
Supplement: Supplementary file 1 [file cancers-16-01106-s001.zip › Supplementary Table S4.pdf]

Surgery type	N	%
**No**	1	0.98
**BSO**	6	5.88
**BSO, OM**	2	1.96
**BSO, OM, LND**	2	1.96
**HBSO**	23	22.55
**HBSO, OM**	14	13.73
**HBSO, OM, LND**	1	0.98
**HBSO, OM, AP**	4	3.92
**HBSO, OM, AP, PPLND**	9	8.82
**HBSO, OM, LN biopsy**	1	0.98
**HBSO, OM, LND**	19	18.63
**HBSO, OM, bladder-involved focus resection**	1	0.98
**HBSO, excision of mesenteric nodules**	1	0.98
**HBSO, omental biopsy**	2	1.96
**HBSO, peritoneal, and omental biopsies**	1	0.98
**HBSO, right hemicolectomy**	1	0.98
**HBSO, OM, AP, PPLND, splenectomy**	1	0.98
**HBSO, OM, AP, PPLND**	1	0.98
**HRSO, OM, AP, PPLND**	1	0.98
**LSO**	4	3.92
**LSO, omental sampling**	1	0.98
**RO**	2	1.96
**RSO**	1	0.98
**RSO, PPLND**	1	0.98
**SO**	2	1.96

Table S4: Details of surgery type

Abbreviations: AP: appendectomy; BSO: bilateral salpingo-oophorectomy; HSBO: hysterectomy bilateral salpingo-oophorectomy; LN: lymph node; LND: lymph node dissection; LSO: left salpingo-oophorectomy; OM: Omentectomy; PPLND: pelvic and para-aortic lymphadenectomy; RO: right oophorectomy; RSO: right salpingo-oophorectomy; SO: salpingo-oophorectomy.
