# Supplementary material for: Malignant Brenner Tumor of the Ovary: A Systematic Review of the Literature
Source: Cancers (Basel). 2024 Mar 9;16(6):1106. doi: 10.3390/cancers16061106 (PMC10968811; doi:10.3390/cancers16061106)
Supplement: Supplementary file 1 [file cancers-16-01106-s001.zip › Supplementary Table S5.pdf]

	Molecular findings
MBT	CDKN2A and CDKN2B inactivating mutations<br>FGFR3 and PIK3CA activating mutations<br>MDM-2 amplification<br>TP53 truncating and missense mutations<br>BAP1 in rare cases<br>BRCA-2 mutation in a single case

Table S5: The summary of the molecular alterations in malignant Brenner tumors
